# Contraception

**DOI:** 10.1186/1472-6874-4-S1-S25

**Published:** 2004-08-25

**Authors:** Sharon McMahon, Lisa Hansen, Janice Mann, Cathy Sevigny, Thomas Wong, Marlene Roache

**Affiliations:** 1 Centre for Infectious Disease Prevention and Control, Health Canada, Tunney's Pasture, Ottawa, Canada; 2 Centre for Infectious Disease Prevention and Control, Health Canada, Tunney's Pasture, Ottawa, Canada; 3 Centre for Infectious Disease Prevention and Control, Health Canada, Tunney's Pasture, Ottawa, Canada; 4 Centre for Infectious Disease Prevention and Control, Health Canada, Tunney's Pasture, Ottawa, Canada; 5 Centre for Infectious Disease Prevention and Control, Health Canada, 400 Cooper Street, Suite 2005, Ottawa, Canada; 6 Centre for Infectious Disease Prevention and Control, Health Canada, Tunney's Pasture, Ottawa, Canada

## Abstract

**Health Issue:**

Contraception choices affect the long-term sexual health and fertility of women and men. Data from the 1998 Canadian Contraception Study and the 2000/2001 Canadian Community Health Survey were assessed for measures of contraceptive use and familiarity with various methods among Canadian women.

**Key Findings:**

The oral contraceptive (OC) pill is the dominant method of contraception for Canadian women. Canadian women demonstrate high awareness of the benefits of condom use, but 75% are unaware of the female condom. Among youth, condom use drops as OC use increases. Contraception use in sexually active females aged 15–17 is fairly high, but use is inconsistent. Sexually active adolescent females report high awareness of emergency contraception but poor knowledge of the time frame within which it is most effective. Women aged 35–44 are more familiar with and likely to choose sterilization than younger women. There has also been a shift away from tubal ligation in favour of vasectomies.

**Data Gaps and Recommendations:**

National data to guide policy and program development are limited. More data are needed on contraception use among males, and factors affecting accessibility, adherence and negotiation of choice. The importance of dual protection, and correct and consistent use of the chosen contraceptive method must be communicated to younger Canadians, as well as health care providers and educators. All women of reproductive age should be made aware of emergency contraception methods and increased efforts on sexual health promotion and education are required. Further research is essential to develop expanded contraceptive choices.

## Background

Contraceptive choices affect the long-term sexual health and fertility of women and men, particularly when contraception is not used correctly or consistently. For many women, the ability to control their fertility has enhanced their ability to control their lives; however, with this power has come a greater responsibility for contraception in a relationship. Given that the majority of contraceptive methods available are made to be used by women and that the consequences of a contraceptive failure can have a greater impact on the life and health of a woman than on her partner, this is a vital issue in women's health. Ideally, in any relationship both partners will discuss the most appropriate method of contraception and be committed to using it correctly and consistently. The context in which men and women make decisions related to contraception has changed with the advent of oral contraception (OC) some 40 years ago and, more recently, with the increased awareness of HIV/AIDS and sexually transmitted infections (STIs).

The decision to use one contraceptive method over another is influenced by personal choice, perceptions of efficacy, personal risk, access, age, cost, gender, education, ethnicity, marital status, current number of children, sexual orientation, pattern of sexual activity and level of co-operation between partners.

The objectives of this chapter are to provide a brief historical perspective of contraception; highlight recent trends in contraceptive use among Canadian women; examine their familiarity with various methods, including emergency contraception; consider the factors that influence choice across life stages and differences in this regard between the sexes; and make recommendations based on this information. The chapter will focus on two of the leading contraceptive methods, OC and the condom.

## Historical Perspective

History has demonstrated that, for centuries, women and men have been using various methods of contraception, some of which were pernicious to women's overall health, to prevent pregnancy and control their fertility[1]. In Canada, by the late 1800s many forms of contraception were being used, including abstinence, vaginal sponges, diaphragms, condoms, withdrawal, douching, prolonged breast-feeding, rhythm method, sterilization and abortion. These methods, now greatly improved, continue to be used. From the late 1860s to the 1930s a significant amount of controversy, conflicting theories and social stigma were associated with contraception usage. Many women were discouraged from seeking information from physicians, who were often reluctant to provide information about contraception[2]. By the 1960s, there was a greater acceptance of contraception, evidenced by the legislative changes in 1969 that legalized birth control. With the advent of the oral contraceptive pill, improved accessibility to other forms of contraception, and later the risk of HIV/AIDS, society's view of contraception has evolved and continues to do so.

## Methods

Cross-sectional data from cycle 1.1 of the 2000–2001 Canadian Community Health Survey (CCHS) were analyzed. Results from the 1998 Canadian Contraception Study (CCS), other surveys and research were examined to provide a picture of current contraceptive practices among women of various ages and the factors that influence their choices. The 1998 CCS compared four national surveys of contraceptive use in Canada: the Canadian Fertility Study (CFS) (1993) and three Canadian Contraception Studies (1993, 1995 and 1998).

## Results

### Methods of Contraception

#### Oral Contraceptive Pill

Introduced in the 1960s, oral contraception is widely used around the world. According to the 1998 Canadian Contraception Study, OC is the dominant method of contraception for women in Canada, used by over 28% of all women and 43% of sexually active women[3]. Like all forms of contraception, there are advantages and disadvantages associated with the use of OC, which should be evaluated by women and their health care provider when this method is being considered. Of particular concern is the fact that it does not provide protection against STIs.

#### Male Condoms

Condoms are an effective method of birth control and currently provide the most reliable barrier method for protection against HIV and STIs[4]. The estimated effectiveness of the male condom, used correctly and consistently, is 88%[5]. According to the CCS, women indicated a high awareness of the condom and, of those who were familiar with the condom, most ranked it as a "very" or "somewhat" favourable method[3].

#### Female Condoms

The female condom, or vaginal pouch, is a relatively new method which gives women control of a barrier method and provides some STI protection. The CCS revealed that only 25% of the women sampled had heard of the method, although 41% of young unmarried women 15 to 17 years of age were aware of it. No one reported using it as a method of contraception, and only 3% had a favourable attitude towards the female condom[3]. Cost is a factor, as the female condom is considerably more expensive than the male condom.

#### Dual Protection

Dual protection is defined as any method of birth control combined with a condom. Dual protection prevents pregnancy and prevents the transmission of an STI, but it should be noted that some STIs, such as human papillomavirus (HPV), can exist outside the vagina or at the base of the penis, and the protection afforded by a condom is not absolute. Particularly important for younger people or those not in mutually monogamous relationships is the fact that dual protection can help maintain good sexual health throughout an individual's lifetime.

Studies on dual method use are limited, but several reasons can be hypothesized as to why concurrent use with a condom appears to be low. First, individuals seem to perceive pregnancy as the greatest imminent threat, and once this has been addressed their motivation to take further measures is low. Second, some methods are compliance-dependent, such as OC or condoms, and others compliance-independent, such as sterilization or the intrauterine device (IUD). Those using compliance-independent methods may be less likely to have condoms available or to take extra measures[6].

#### Emergency Contraception

Often referred to as the "morning after" pill, emergency contraception (EC) has been available for more than two decades in North America and Europe, offering an effective choice after unprotected sex or contraception failure, or for use in cases of sexual assault. EC is highly effective in preventing pregnancy if taken within 72 hours of unprotected sex. It works by either preventing ovulation or changing the lining of the uterus so that implantation cannot occur. EC will not work if a woman is already pregnant[7].

A survey conducted by Langille and Delany of 14- to 19-year-olds at a high school in Nova Scotia showed that 80% of the girls had heard of EC, but only 10% were aware of the time limits within which EC is effective. Overall, they had a poor understanding of the risks, benefits or effectiveness of EC. These young women had learned about EC for the most part through their sexual health education classes in grades 7 to 9. Only 2% had heard about it through their physician[8].

The challenge facing policy makers is to raise awareness about the potential role of EC in women's reproductive health. The cost of medical care associated with an induced abortion is estimated to be $618, whereas the cost of a physician visit and prescription for EC is approximately $32.27[9]. This simplistic comparison ignores the large social and psychological costs of abortion or unintended pregnancy.

#### New Methods

There are some new contraceptive methods on the horizon but not yet available in Canada. Vaginal ring and injectable monthly contraceptives are combined estrogen and progestin methods that are highly effective and, because they do not require a daily ritual, may improve compliance. Progesterone-releasing intrauterine devices and implants offer effective choices for women for whom estrogen is not recommended[10].

Another new option for Canadian women is the contraceptive patch, approved for use in Canada in August 2002. It works in the same way as OC, but in the form of a once-a-week patch. The patch, which is the size of a match-box, is worn for three consecutive weeks, and then a patch-free week follows.

Expectations for a male pill in the near future have been lowered, as clinical trials in China funded by the World Health Organization (WHO) resulted in several serious side effects. This trial has been stopped, but research into other options does continue[11]. Another clinical trial, and a more promising one, is the use of injections that combine testosterone and androgen-progestogen. Again initiated by the WHO, the study involves nearly 200 participants and attempts to determine the acceptability of these hormonal methods by men and women. This could prove to be an acceptable option for couples in monogamous relationships, for whom contraception and the consequences of failed contraception are shared; however, it may not be an advisable option for women at risk or those with multiple partners[12].

Research into microbicides, which can kill viruses, bacteria and fungi, and sperm, is under way, though it will likely be many years before these precautionary measures become widely available. The potential of an insertable gel (also referred to as the "invisible condom") in combatting HIV and STI is huge and would give women control over both pregnancy and STI prevention. There has been limited investment by pharmaceutical companies, only 1% of funding for microbicides coming from the private sector. Microbicides will be particularly valuable for women in abusive relationships, for sex trade workers or for those with partners involved in outside relationships[13].

#### Choice of Contraceptive

**Figure 1:**
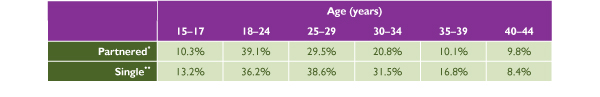
**Percentage of Women Reporting Oral Contraceptive Use in Previous Month **[14]. Source: Canadian Community Health Survey, Statistics Canada, 2000–2001

Analysis of CCHS data indicates that 17.7 % of the women aged 12 to 50 who were surveyed reported using OC when asked about medications taken in the previous month. It is important to note that the module on medication in this survey was asked only in Ontario, and the question set referred to all medications, not only contraceptives. Women between the ages of 18 and 29 reported the highest usage of OC, and single women reported higher usage, as shown in Figure 1[14].

The CCHS results shed light on factors other than age that affect OC use. Women in the low to low-middle income range were less likely to use OC in the previous month than their higher-income counterparts. Rural versus urban living had no impact on usage, but partnered women (married or common-law) reported significantly lower usage rates, 14.7 % versus 21.4% (*p *< 0.001) among single women. Women who described themselves as "white" were significantly more likely to use OC than those from other racial or cultural backgrounds (20.3% versus 9.5%, *p *< 0.001). Finally, a correlation between OC usage and education was also observed, in that women who had graduated from high school or had any post-secondary education were significantly more likely to use OC than women with less than a high school diploma (20.3% versus 9.2%, *p *< 0.001)[14].

The 1998 CCS indicated that the most notable shift in choice of contraceptive method in recent years is related to sterilization, with a move away from tubal ligation and an increase in vasectomy. Female sterilization was reported by 24% of respondents in 1984, a proportion that dropped to 10% in 1998. Over the same period, the proportion of male sterilization increased from 9% to 14%[3]. Martin and Wu noted that the rate of sterilization in Canada is high as compared with other industrialized countries[15].

In addition, the results provide a strong indication that women were better informed in 1998 than in 1984 about protecting themselves against STIs. Condom use has increased significantly, from 6.2% in 1984 to 21% in 1998, and women's overall knowledge has improved regarding the inability of OC to provide protection against STIs. In 1998, 3% of women indicated their erroneous belief that OC protects against STIs, down from 6% in 1993[3].

While Canadian women have maintained high familiarity ("Familiarity" in this study indicates that a woman knew enough about the method to be able to consider its suitability for her as a method of contraception). with OC and condoms over time, their familiarity with other methods, such as sterilization, withdrawal, the diaphragm, the IUD and the rhythm method, has steadily declined since 1984[3]. This may be, in part, because media discussions on contraception are often heavily focused on OC and the condom. The authors of the CCS stress that familiarity with the wide range of contraceptive options is necessary for women if they are to make the best choices throughout their lives. In the CCS, 9% of women reported using withdrawal as their method of contraception, a choice that is neither highly effective nor protective against STIs. The rate was highest among unmarried women: 22% of those 15 to 17 years of age, 11% of those 18 to 34, and 15% of those 35 to 44 relied on this method. Injectable progesterone, also known as Depo-Provera is a hormonal method that was approved for use in Canada in 1997. Only 21% of women were familiar with Depo-Provera, and less than 1% were usingit[3].

Finally, the percentage of women who were not using any method of contraception increased from 21% in 1984 to 25% in 1995, a finding that may be explained by the aging of the population[3].

### Contraception through Various Life Stages

#### Adolescents

Contraception use at sexual debut among women in the 15 to 17 age group is quite high, 80% reporting its use the first time they had intercourse, according to the CCS. While the sample was small and did not allow for testing of statistical significance, it provides an indication that many adolescents are using contraception at sexual debut. With regard to consistency of use over the previous six months, however, only 60% of unmarried teens 15 to 17 years of age reported always using contraception and, as noted earlier, 22% of this sexually active group rely on withdrawal[3].

The rate of teenage pregnancy in Canada remains high. A Statistics Canada report estimated that in 1997 the number of women aged 15 to 19 who had given birth was 19,724, and the number who had had an induced abortion was 21,233[16]. Although this is a multi-faceted issue, comprehensive sexual health promotion and education would go a long way towards reducing unintended pregnancies in teenagers.

Canadian studies show that rates of condom use drop as use of OC increases among youth, especially with a main partner[17]. Condom use tends to be higher when there is a perceived risk of both becoming pregnant and acquiring an STI. For those in casual relationships condom use is higher, regardless of hormonal contraceptive use, suggesting that adolescents view these relationships as carrying a higher risk of STI transmission. Prevention and education programs need to address relationships with a main partner to improve the use of dual protection[18]. Further, adherence to the method used is critical to its effectiveness, and this is another aspect of sexual health education on which an increased focus would be beneficial. The 1988 Canada Youth and AIDS Study indicated that adolescents may not necessarily continue with their method of contraception if their relationship breaks up or if intercourse is infrequent[19].

Aboriginal youth, street youth, sex trade workers and other marginalized youth are not represented in these studies, and although they are small in number they may be at higher risk of both unintended pregnancy and STIs. More targeted research is needed to guide program development and to ensure that, once developed, programs meet the needs of these Canadian youth in ways that are appropriate, accessible and culturally sensitive.

#### Women Aged 18 to 29

Women in the 18 to 29 age group are often completing their studies, establishing themselves in careers, and entering into long-term relationships while at the peak of their fertility. It follows that this group has the highest usage of contraception. The popularity of methods in this age group, in descending order, are OC, condom, IUD, diaphragm, foam, rhythm and withdrawal, and "other." Although the popularity of OC has changed very little, use of the condom was higher in 1995 than 1984, indicating that safer sex messages are having some effect[15].

Fisher and Boroditsky examined the sexual activity and contraceptive choices of single Canadian women aged 15 to 17, 18 to 24, and 25 to 29. These women are aware of and have positive opinions about OC and condoms, but their awareness and opinions on other methods, such as the female condom, emergency contraception or injectable methods, were limited and not favourable. These young women reported a high level of contraceptive use at first and last intercourse with a current partner, but over time they were less likely to continue dual protection and may have used less reliable methods, such as withdrawal. The authors suggested that efforts should be made to encourage consistent use of appropriate contraceptive methods over time[20].

A 1999 U.S. study examined the differences in perceptions and priorities of men and women aged 20 to 27 with regard to contraception. Women, particularly married women, ranked the prevention of pregnancy as most important. STI prevention and absence of associated health risks followed or were viewed as equally important. Men ranked pregnancy and STI prevention equally as the most important. Single men and women ranked the importance of STI protection higher than the married group. The differences between the perceptions of the sexes could be attributed to four factors: (1) women have a higher exposure to information about contraception, as it is often considered the domain or responsibility of women; (2) women tend to have more experience in making choices about contraception use for the same reason; (3) the actions required by men and women vary depending on the method used; and (4) men and women do not perceive the risk of an unwanted pregnancy or STI equally[21].

Men and women agreed in their ranking of OC and the condom as to ease of use and spontaneity. They did not, however, agree on the method that most interfered with pleasure: women identified the diaphragm and men the condom[21].

#### Women Aged 35 to 44

According to the CCS, women aged 35 to 44 reported a higher level of familiarity with sterilization than younger women and lower familiarity with reversible methods. This group held a much more positive attitude about male and female sterilization and the IUD. Although women aged 35 to 44 viewed OC quite positively, there was some indication that they were poorly informed about its risks and potential long-term benefits, particularly with the arrival of low-dose oral contraception, which allow many women to use this method for longer than traditional oral contraception[3].

When asked about the method used, single women relied on the pill and condom, and married women were most likely to rely on sterilization, of themselves or their partners: 23% of married women aged 35 to 44 reported using sterilization as their method of contraception, 10% of themselves and 14% of their partners. A change in contraceptive method is common in this group, with the switch to sterilization reflecting the desire for a permanent contraceptive method. Twenty-three percent of married women whose previous method had been the pill changed methods on their physician's advice[3].

A study conducted in the United States examined two U.S. data sets on sterilization, Cycle 5 (1995) of the National Survey of Family Growth and the 1987–1988 National Survey of Families and Households, to analyze why tubal ligation is so much more common than vasectomy. The authors concluded that some individuals make a decision to be sterilized without considering sterilization options for their partners, and this is more likely if they are unmarried. Factors that influence the decision include marital status, and surprisingly high numbers of unmarried women seek sterilization. Also, there are low rates of vasectomy among men in cultural minority groups and high levels of tubal sterilization among women in these groups. Age and current number of children at last birth play a role in the decision. Education is also a factor, given that the higher the woman's educational level the less likely it is that a couple will opt for tubal ligation and the more likely that they will choose vasectomy, whereas couples with the same level of education will more likely choose tubal ligation Finally, religion affects choice, given that fewer Catholics undergo vasectomy or tubal ligation than Protestants[22].

Canadian women indicate that they would prefer their partner to undergo a vasectomy, and for many reasons. The male procedure is simpler and has fewer risks: a 15-minute office procedure under local anaesthetic versus a 30-minute surgical procedure under general anaesthetic[3].

## Discussion

### Data Limitations

Despite the availability of excellent data from the CCHS, the 1998 CCS and Canadian fertility studies that are frequently cited in this chapter, there are limited Canadian data on contraceptive use by women of all ages and even fewer on contraceptive use by men. More research is needed on accessibility, adherence, cause of unintended pregnancy, and negotiation of choice and use of contraception with one's main partner in order to provide a clearer picture of contraceptive use in Canada today. A further limitation is the lack of data on Aboriginal people, street youth, sex trade workers and other marginalized populations.

### Policy Implications and Recommendations

Although there are more choices of effective methods of contraception available to Canadian women today than in the past, research shows that not all women have the same level of familiarity with or access to the various methods. Messages about the importance of correct use and adherence to the contraceptive method chosen are a vital component of the information needed by women. Until such time as there are more effective options of contraception available to men, women need to remain diligent to ensure that the method used provides the level of protection required. The challenge facing policy makers and program administrators will be to increase awareness and knowledge among women of all ages to help them make the best contraceptive choices throughout their lives.

Consideration of contraceptive methods should be balanced between the objectives of reducing unintended pregnancies and the need for STI protection. Before the pandemic of HIV, the focus of policy makers in this area was population growth, but given the serious consequences of an STI, contraception has the potential to offer broader protection for women and men.

The following recommendations are made:

• Increase efforts in sexual health promotion and education to raise awareness in Canadian women of all ages about the advantages and disadvantages of the various contraceptive methods available to them.

• Heighten awareness of the importance of dual protection, particularly in younger women and men as well as in physicians, health care providers and educators. Women place higher emphasis on pregnancy prevention than STI prevention, which may be a result of lack of information.

• Adherence to the contraceptive method chosen is critical to the effectiveness that it offers. Counselling concerning the choice of contraception method must take into account the individual needs of each woman and methods that require co-operation from a partner may not be appropriate for all women.

• Raise awareness of access to emergency contraception in women of all ages. This should include the time frame for which EC is effective and information on where EC can be accessed.

• Increase resources to fund research and trials associated with new, reversible contraceptive methods for men and the development of safe and effective microbicides.

## Notes

The views expressed in this report do not necessarily represent the views of the Canadian Population Health Initiative, the Canadian Institute for Health Information or Health Canada.

## References

[B1] Walby S (1990). Theorizing patriarchy.

[B2] Mitchinson W (1991). The nature of their bodies: women and their doctors in Victorian Canada.

[B3] Fisher WA, Boroditsky RBM (1999). The 1998 contraception study. Can J Human Sexuality.

[B4] Patrick DM, Wong T, Jordan R (2000). Sexually transmitted infections in Canada: recent resurgence threatens national goals. Can J Human Sexuality.

[B5] Hatcher RA (1989). Contraceptive Technology: International Edition Atlanta GA: AIDS Education Global Information System, National Library of Medicine.

[B6] Cates W, Holmes KK (1999). Contraception, contraceptive technology and STDs. Sexually transmitted diseases.

[B7] (2000). Society of Obstetricians and Gynaecologists of Canada. Sex sense – Canadian contraception guide Ottawa: Society of Obstetricians and Gynaecologists of Canada.

[B8] Langille DB, Delaney ME (2000). Knowledge and use of emergency postcoital contraception by female students at a high school in Nova Scotia. Can J Public Health.

[B9] Trussell J, Wiebe E, Shochet T, Guilbert E (2001). Cost savings from emergency contraceptive pills in Canada. Obstet Gynecol.

[B10] Shvarts S, Kea B (2002). New advances in contraception. Insights.

[B11] Hartmann B (1995). Reproductive rights and wrongs: the global politics of population control.

[B12] Ringheim K (1996). Whither methods for men? Emerging gender issues in contraception. Reproductive Health Matters.

[B13] Reid R Making whoopee over the gel condom. The Ottawa Citizen.

[B14] Beland Y (2002). Canadian Community Health Survey – methodological overview. Health Rep.

[B15] Martin K, Wu Z (2000). Contraceptive use in Canada: 1984–1995. Fam Plann Perspect.

[B16] Dryburgh H (2000). Teenage pregnancy. Health Rep.

[B17] Thomas BH, DiCenso A, Griffith L (1998). Adolescent sexual behaviour: results from an Ontario sample. Part II: adolescent use of protection. Can J Public Health.

[B18] Ott MA, Adler NE, Millstein SG, Tschann JM, Ellen JM (2002). The trade-off between hormonal contraceptives and condoms among adolescents. Perspect Sex Reprod Health.

[B19] King AJC, Beazeley RP, Warren WK, Hankins CA (1988). Canada youth and AIDS study.

[B20] Fisher WA, Boroditsky R (2000). Sexual activity, contraceptive choice, and sexual and reproductive health indicators among single Canadian women aged 15–29: additional findings from the Canadian Contraceptive Study. Can J Human Sexuality.

[B21] Grady WR, Klepinger DH, Nelson-Wally A (1999). Contraceptive characteristics: the perceptions and priorities of men and women. Fam Plann Perspect.

[B22] Bumpass LL, Thomson E, Godecker AL (2000). Women, men, and contraceptive sterilization. Fertil Steril.

